# Supplementary material for: Empirical methods for controlling false positives and estimating confidence in ChIP-Seq peaks
Source: BMC Bioinformatics. 2008 Dec 5;9:523. doi: 10.1186/1471-2105-9-523 (PMC2628906; doi:10.1186/1471-2105-9-523)
Supplement: Additional File 1 — A variety of html documents from the USeq web site detailing the available applications, their best usage, output file type descriptions, command line menus, etc. [file 1471-2105-9-523-S1.zip › USeqUserGuides/JavaDoc.zip!/JavaDoc/bioroot/SearchHit.html]

SearchHit


|  |  |  |
| --- | --- | --- |
| **Overview** \| **Package** \| **Class** \| **Use** \| **Tree** \| **Deprecated** \| **Index** \| **Help** | |  |
| **PREV CLASS**   **NEXT CLASS** | **FRAMES**    **NO FRAMES**     **All Classes** |  |
| SUMMARY: NESTED \| FIELD \| CONSTR \| METHOD | DETAIL: FIELD \| CONSTR \| METHOD |  |


---


## bioroot Class SearchHit

```
java.lang.Object
  bioroot.SearchHit
```

**All Implemented Interfaces:**: java.lang.Comparable

---

``` public class SearchHit extends java.lang.Object implements java.lang.Comparable ```

Holds info relating to a search hit from a database LIKE query.
Word and columnText are lowercasee

**Author:**
:   nix

---

**Constructor Summary**

`SearchHit(java.lang.String columnName,
java.lang.String word,
int id,
java.lang.String columnText,
boolean searchForWordBoundaryMatches)`


| **Method Summary** | |
| --- | --- |
| `int` | `compareTo(java.lang.Object obj)`             Sorts by score |
| `java.lang.String` | `getColumnName()` |
| `java.lang.String` | `getColumnText()` |
| `int` | `getId()` |
| `PatternMatch[]` | `getLooseMatches()` |
| `int` | `getScore()` |
| `java.lang.String` | `getWord()` |
| `PatternMatch[]` | `getWordBoundaryMatches()` |
| `void` | `matchIt(boolean searchForWordBoundaryMatches)` |
| `void` | `scoreIt(boolean searchForWordBoundaryMatches)`             Weighting boundary matches as a score of 5, loose matches as 2. |
| `java.lang.String` | `toString()` |

**Methods inherited from class java.lang.Object**

`equals, getClass, hashCode, notify, notifyAll, wait, wait, wait`

**Constructor Detail**

### SearchHit

```
public SearchHit(java.lang.String columnName,
                 java.lang.String word,
                 int id,
                 java.lang.String columnText,
                 boolean searchForWordBoundaryMatches)
```


**Method Detail**

### toString

```
public java.lang.String toString()
```

:   **Overrides:**: `toString` in class `java.lang.Object`

---


### compareTo

```
public int compareTo(java.lang.Object obj)
```

:   Sorts by score

    :   **Specified by:**: `compareTo` in interface `java.lang.Comparable`

---


### matchIt

```
public void matchIt(boolean searchForWordBoundaryMatches)
```

---


### scoreIt

```
public void scoreIt(boolean searchForWordBoundaryMatches)
```

:   Weighting boundary matches as a score of 5, loose matches as 2.

---


### getId

```
public int getId()
```

---


### getWord

```
public java.lang.String getWord()
```

---


### getColumnName

```
public java.lang.String getColumnName()
```

---


### getColumnText

```
public java.lang.String getColumnText()
```

---


### getLooseMatches

```
public PatternMatch[] getLooseMatches()
```

---


### getWordBoundaryMatches

```
public PatternMatch[] getWordBoundaryMatches()
```

---


### getScore

```
public int getScore()
```


---


|  |  |  |
| --- | --- | --- |
| **Overview** \| **Package** \| **Class** \| **Use** \| **Tree** \| **Deprecated** \| **Index** \| **Help** | |  |
| **PREV CLASS**   **NEXT CLASS** | **FRAMES**    **NO FRAMES**     **All Classes** |  |
| SUMMARY: NESTED \| FIELD \| CONSTR \| METHOD | DETAIL: FIELD \| CONSTR \| METHOD |  |


---
